# Benefit assessment of soil and water conservation from cropland to forest in hilly Loess Plateau at Qinghai

**DOI:** 10.1186/2193-1801-2-S1-S7

**Published:** 2013-12-11

**Authors:** Chuanchuan Zhao, Ninggui Yang, Zhen Wang, Sili Liu, Xu Dong, Wenrong Xin

**Affiliations:** College of Resources & Environment, Shaanxi University of Science and Technology, Xi’an, China; Qinghai Provincial Forest Inventory and Planning Institute, Xi’ning, China

**Keywords:** hilly loess plateau, conversion of cropland to forest, soil and water conservation

## Abstract

The information of slope and vegetation coverage of the monitoring region were extracted, based on DEM (Digital Evaluation Model) and Spot5 Satellite data images, and fishnet grid was generated using GIS (Geographic Information System) and RS (Remote Sensing) technique. Applying the information of slop and vegetation coverage layers into the corresponding space grid by using the function of zonal statistics and analysis, it can realize overlay analysis based on Standards for Classification and Gradation of Soil Erosion (SL190-2007), and obtains the map of soil erosion intensity of the monitoring region. Finally, according to Specifications for Assessment of Forest Ecosystem Services (LY/T1721-2008) and monitoring data of typical plot, the soil and water conservation value from cropland to forest was evaluated quantitatively in 2009. The results showed that the area, on and below the moderate level, was 93600 ha, taking up 50.03% of total conversion of farmland to forest area (185100 ha), which indicates a 14.64 million (t/a) of soil conversion, and a 1520 million Yuan for erosion control. The results of the study showed that the soil and water conservation was very effective.

## Introduction

Soil and water conservation is one of the six most significant projects that helps to keep the high-speed development of our nation's forest industry, but also one of the world's most important ecological projects, taking up a unique place in both the nation's ecological construction and the world's [1]. The project is especially aiming at recovering the vegetation coverage, reducing soil and water erosion, and improving the ecological environment. It, at the same time, readjusts the structure of rural industry, increases farmer income, and fulfill the propose of forbidding further cultivation, which will easily lead to soil and water erosion and land desertification, of cropland step by step [2, 3]. Not only does the project of turning cropland to forest mitigate the severe situation of soil and water erosion, promote the conserving ability of water resources, improve the environment along the Yangtze river and Huang river, and strengthen the region's ability to prevent the occurring of floods and droughts, thus improving the productivity of the land, but also serves as the middle-lower region and encourages the development of industrial and agricultural production, while at the same time serves as the firm foundation of sustainable development of our society.

The hilly loess plateau of Qinghai province, which is located at between the loess plateau and Qinghai-Tibet Plateau, is the typical drought and hemi-drought region and has a relatively high elevation of which the ecological system is a little bit fragile, and the population density is huge. Additionally, the high index of cultivation and the soil and water erosion is too serious to threaten human society. As the most important practical and experimental region of Qinghai province's conversion of cropland to forest project, this region has accomplished the target area of 0.1851 million ha from 2000 to 2009.

The benefits gaining from the project of conversion from cropland to forest is what the society is concentrating most about. Although there is little literature hitherto containing evaluation modeling of conserved land [1], forest land formed by the conversion of cropland to forest is part of the forest resources, therefore, the study quantitatively evaluates the benefit of the region's soil and water conservation through the combination of microscopic analysis and field monitoring, based on the method to assess the effectiveness of soil and water conservation of the conversed land, and finally providing a persuasive basis for further establishment of soil and water conservation and selecting well-matched plants for future conversion.

## Study area

Located in the loess hilly and gully region of Qinghai province, the researched area boarders Datong River on the north, Xiqin Mountain on the south, Riyue Mountain on the west, and stretches straight forward to the borderline on the east. It has a total area of 3.451 million ha, including Xining city, north, south, and east states of Qinghai, and Huangnan state, adding up to 14 counties in all, which make up 10% of the total area of Qinghai province and 70% population along with the industrial and agricultural products. In addition, the GDP of this region is up to 55% of that of the province

**Figure 1:**
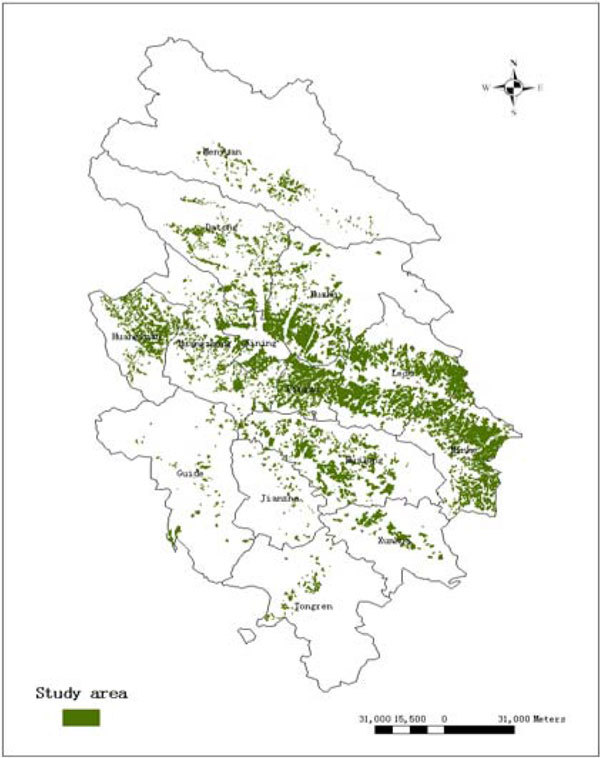
**Map of the distribution of the conserved land**.

According to the latest survey of type II statistics of the forest industry, the distribution information of every county in the monitoring region is showed in Figure 1.

## Methods and data resources

### Methods

Based on GIS/RS technology, which connects with net grid already built by extracting information concerning the slope, land use scenario, and vegetation coverage factors of the monitoring region, the research calculated these data to build layers of soil erosion intensity through zonal statistics according to Standard SL190-2007.

### Data derivation

Landform information is based on the DEM system that generates a 1:50,000 electronic topographic map of the loess hilly and gully region. The classification of the used land is based on type II information on the latest periodical of forest industry, which indicates that the information is gained by the statistical data of the forests engineering to build up a well established image of the conserved land. The information of the vegetation coverage factor derives from the revised Spot5 Satellite image data (2007-08) with 10 meter full-color resolution. The background information of water and soil erosion (erosion modulus and soil bulk density) and the effectively proved results of microscopic monitoring were built on the observation and surveys of the representative conserved model land of the loess hilly and gully area.

## Evaluation

### Erosion intensity

#### Factor of slope

The slope of certain point is the crossing angle between the horizontal ground and the tangent line through the cut point. The slope value can be calculated based on the command of Natural Neighbor Interpolation and DEM grid to analysis related data [4].

**Figure 2:**
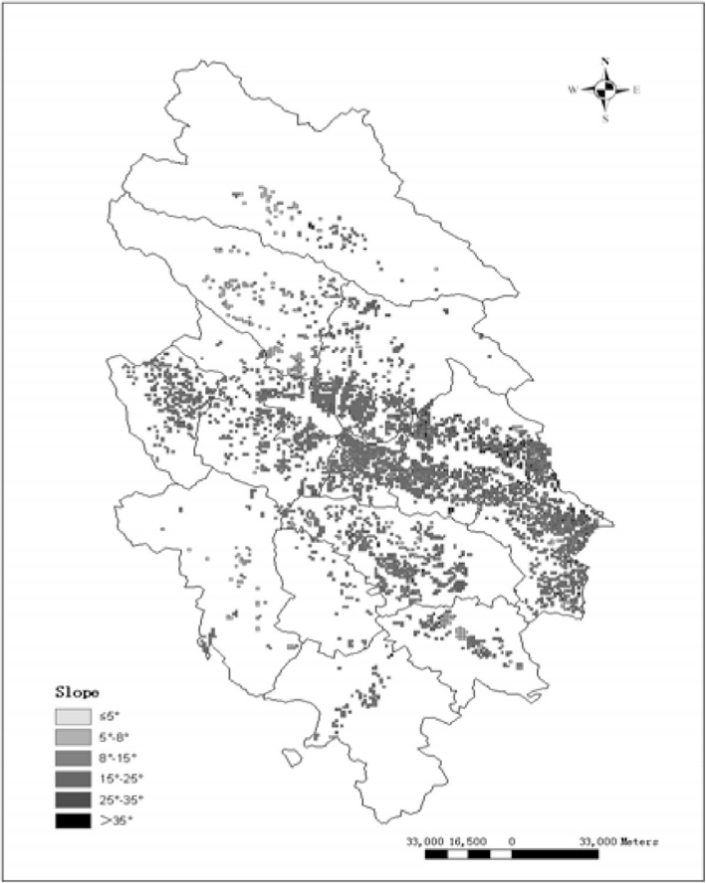
**The map of slope grade**.

DEM can be established with 10 meters resolution of the researched region, based on ArcGIS9.0 program and 1:50,000 electronic topographic map of the landform. Also, AcrGIS9.0 has the command to pick out slope information by using the Slope tool of Spatial Analysis program. Once the slope is generated, the program can divide the slope into six grades that are ≤5°, 5°-8°, 8°-15°, 15°-25°, 25°- 35°, and <35° by using classification statistics command to create a file containing slope information, which is achieved by Reclass Function according to Standard SL190-2007. The results of the slope grades can be seen in Figure 2.

#### Factor of vegetation coverage

**Figure 3:**
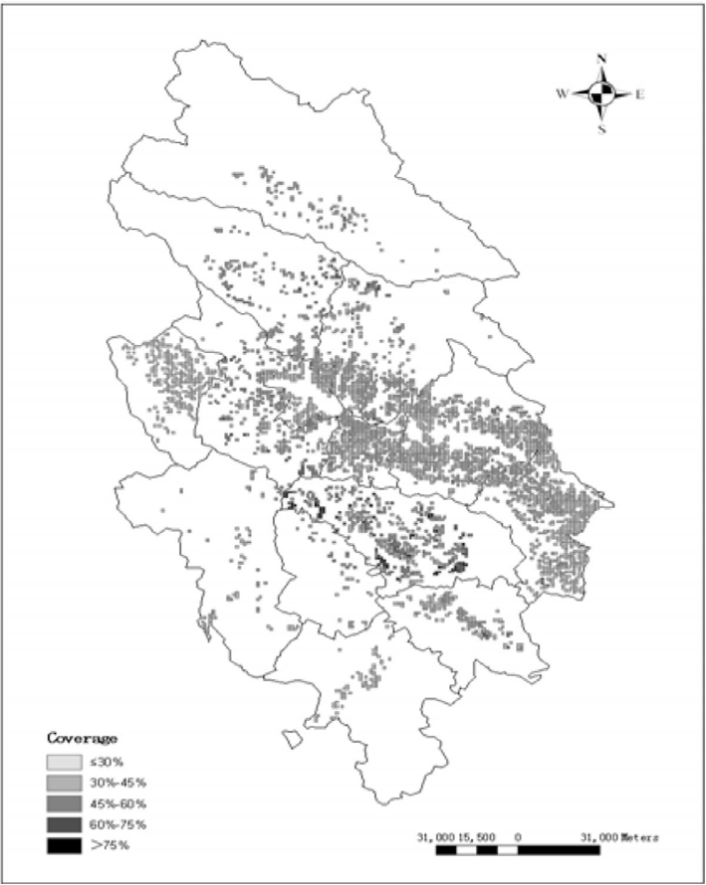
**The map of vegetation coverage grade**.

Vegetation coverage is defined as the percent of vertical projective area per unit area with obvious special and temporal variation characters [5]. The information of vegetation coverage can be detected, according to the combination of high contrast image produced by Spot5 Satellite image with 10 meters resolution and the real field survey. Researched area can be divided into five grades that are ≤30%, 30%~45%, 45%~60%, 60%~75%, <75% respectively based on Standard SL190-2007. The result of vegetation coverage can be seen in Figure 3.

### Fishnet establishment and factors analysis

Regional grid, as a critical tool of data organization, is essential to the application and realization of all the analytical factor layers [5]. The information of slope and vegetation coverage that is utilized in proceeding regional monitoring of erosion is vector data. Because of the complex data structure of vector polygon, when regional superposition is analyzed there can be many polygon scraps which make it hard to conduct other operations [6]. Using the expand section of fishnet in ArcGIS9.0, many grids with rows and columns can be generated to better resolve the problem and offer more convenient function of overlay analysis and property operations as well as updating data in time.

It is critical to determine the resolution of the grids, and in order to avoid unnecessary visual memory, grids of proper resolution will lead to satisfactory results [7, 8]. The researched region has a total area of 3.514 million ha, so the grid is set to 1000 × 1000 m type and it can generate grids with 350 rows and 200 columns. 200 resolution raster is applied to slope and vegetation coverage, with the demand of applying the research into practice. Area dominate methods is used in slope and vegetation coverage is measured by average value, the results of which are assigned to the grids of monitoring regions.

### Benefit evaluation

#### Monitoring results

**Figure 4:**
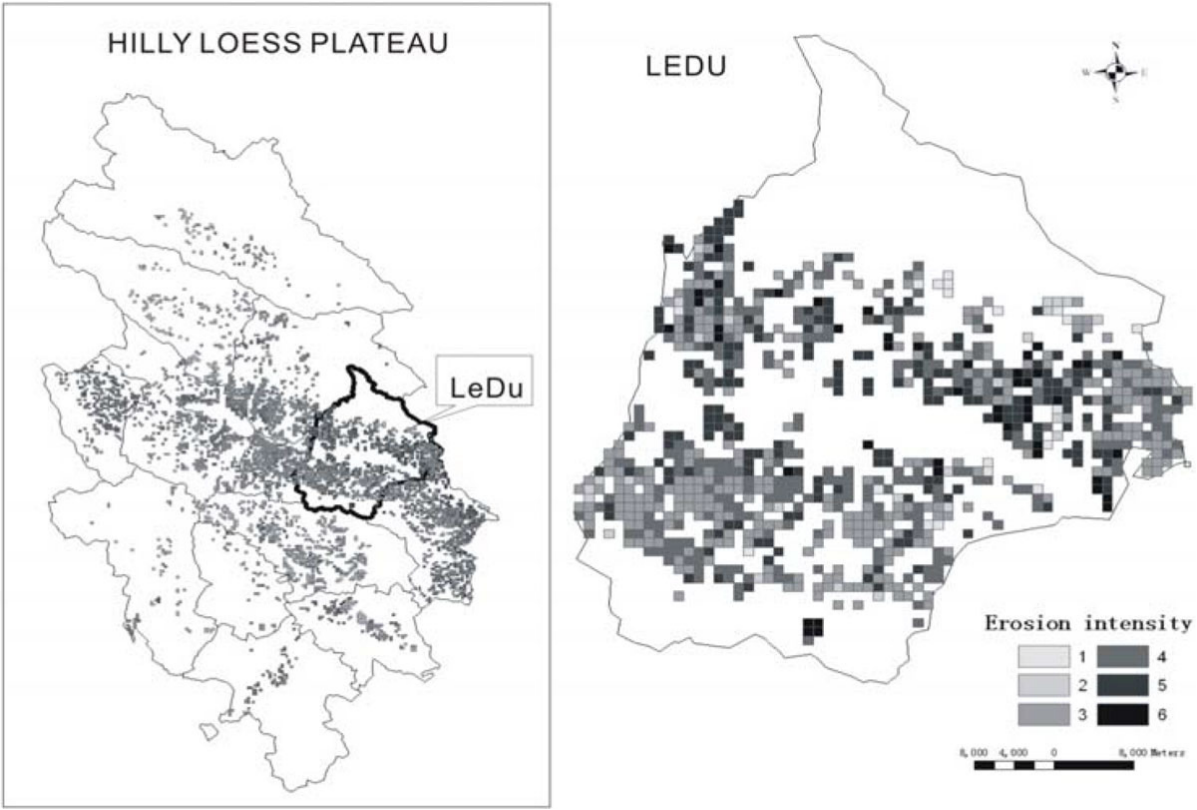
**Map of erosion intensity grades distribution**.

Based on above analysis, the soil erosion intensity of the researched area is divided into 6 grades of slight, gentle, moderate, strong, extremely strong and intense. According to the standards and methods mentioned above, area and percent of different type of erosion is listed in Table 1, and soil erosion intensity level can be seen in Figure 4.

**Table 1:** The area of variety erosion intensity of the monitoring area

Erosion grades	Grids	Erosion area (km^2^)	Percent (%)
Slight	114	114	6.16
Gentle	82	82	4.43
Moderate	730	730	39.44
Strong	588	588	31.77
Utmost	279	279	15.07
Severe	58	58	3.13

According to Table 1, on and below moderate soil erosion level of the monitoring region of conserved land area is 93.6 thousand ha, making up 50.03% of the total area, with 58.33% and 4.89% area of slop ≥15°and slope ≥25°respectively.

#### Result validation

The actual erosion intensity can be determined by quadrat survey of the researched area after field research. Picking out the same place to validate the effectiveness of the monitoring results, and the results can be seen in Table 2.

**Table 2:** Validation results of soil erosion intensity

Number	County	Forest age	Results of monitoring	Results of fields
Plot 1	Tongren	9	Utmost	Utmost
Plot 2	Ledu	9	Moderate	Moderate
Plot 3	Ledu	9	Moderate	Strong
Plot 4	Ledu	9	Gentle	Gentle
Plot 5	Ledu	9	Gentle	Gentle
Plot 6	Ledu	7	Moderate	Moderate
Plot 7	Pingan	9	Moderate	Moderate
Plot 8	Pingan	9	Moderate	Gentle
Plot 9	Pingan	4	Gentle	Gentle
Plot10	Pingan	9	Gentle	Gentle
Plot11	Pingan	4	Moderate	Strong
Plot12	Jianzha	7	Moderate	Moderate
Plot13	Minhe	7	Moderate	Strong
Plot14	Menyuan	4	Gentle	Gentle
Plot15	Datong	1	Strong	Strong

Comparing soil monitoring results of the researched region and field survey results, it can be discerned that the general tendency of the two results is matched consistently. The fifteen sample lands, except for four inconsistent samples, Plot 3, Plot 8, Plot 11, and Plot 13, are all in good relationship with the monitoring results and have high consistency of 73.33%.

#### Evaluation

According to the Evaluation in Project for Construction of Conservation of Cropland to Forest (GB/T23233-2009), the evaluation methods of regional conserved land is based on microscopic monitoring of regional water and soil erosion of the conserved land and the monitoring results of typical plots. According to Jiao juying et al., the soil loss tolerance of the Loess Plateau ranges from 2800 to 5100 t/(km^2.^a) [9], which shows that the conserved lands are effective in preventing soil erosion if its intensity of the conserved land of loess hilly area is on or below the moderate level. In addition, Specifications for Assessment of Forest Ecosystem Services (LY/T1721-2008) can be referred in quantitative evaluation of the conserved lands.

The average erosion modulus of the observed 18 erosion gully in the nearest 2 years will be taken as the modulus for non-forest soil erosion, the area of which is 20100 t/km^2.^a. At the same time, the average value, 1.22 t/m^3^, of the sample soil collected from 47 conserved lands is used as the value of the soil bulk density. According to the Standards SL190-2007, the value of slight, gentle, and moderate level of erosion modulate is 1000 t/km^2.^a, 2500 t/km^2.^a, 5000 t/km^2.^a respectively. Soil fixation and its benefit can be calculated and evaluated according to Specifications LY/T1721-2008. The results are presented in Table 3.

**Table 3:** Evaluation of water and soil conservation

Erosion grades	Slight	Gentle	Moderate	Total
Amount of soil fixation (million t/a)	2.18	1.44	11.02	14.64
Benefit of soil fixation (million Yuan)	230	150	1140	1520

In Tables 1 and 3, the area of conserved lands, on and below moderate level, of soil erosion is up to 93.6 thousand ha, taking up 50.03% of the total area (185.1 thousand ha) of conserved lands, and the amount of fixing soil is 14.64 million t/a with a benefit of 1520 million Yuan, which indicates that the water and soil conservation of the conversion of cropland to forest is very effective.

## Conclusion

The area, on and below the moderate level, of soil erosion intensity of the conserved land is 50.03% of the total monitoring area, and among them, 53.44% of which has a slope of 15°~25°, while 49.65% of which has more than 30% vegetation coverage, which make great contribution to water and soil conservation of the conserved land of monitoring areas.

The amount of fixing soil of the conserved land of the monitoring area is 14.64 million (t/a) with a benefit of 1520 million Yuan, which indicates that the water and soil conservation of the conversion of cropland to forest is very effective.
